# Scans for signatures of selection in Russian cattle breed genomes reveal new candidate genes for environmental adaptation and acclimation

**DOI:** 10.1038/s41598-018-31304-w

**Published:** 2018-08-28

**Authors:** Andrey A. Yurchenko, Hans D. Daetwyler, Nikolay Yudin, Robert D. Schnabel, Christy J. Vander Jagt, Vladimir Soloshenko, Bulat Lhasaranov, Ruslan Popov, Jeremy F. Taylor, Denis M. Larkin

**Affiliations:** 1 The Federal Research Center Institute of Cytology and Genetics, The Siberian Branch of the Russian Academy of Sciences (ICG SB RAS), 630090 Novosibirsk, Russia; 2 Institute of Biodiversity, Animal Health and Comparative Medicine, University of Glasgow, Glasgow, G12 8QQ UK; 3 Agriculture Victoria, AgriBio, Centre for AgriBioscience, Bundoora, 3083 Victoria Australia; 4 School of Applied Systems Biology, La Trobe University, Bundoora, 3083 Victoria, Australia; 5 Division of Animal Sciences, University of Missouri, Columbia, MO 65211-5300 USA; 6 Siberian Research Institute of Animal Husbandry, 630501 Krasnoobsk, Russia; 7 Shuluuma IAPC, Kizhinga, 671450 Buryatia, Russia; 8 Yakutian Research Institute of Agriculture, 677001 Yakutsk, Russia; 9 Royal Veterinary College, University of London, NW01 0TU London, UK

## Abstract

Domestication and selective breeding has resulted in over 1000 extant cattle breeds. Many of these breeds do not excel in important traits but are adapted to local environments. These adaptations are a valuable source of genetic material for efforts to improve commercial breeds. As a step toward this goal we identified candidate regions to be under selection in genomes of nine Russian native cattle breeds adapted to survive in harsh climates. After comparing our data to other breeds of European and Asian origins we found known and novel candidate genes that could potentially be related to domestication, economically important traits and environmental adaptations in cattle. The Russian cattle breed genomes contained regions under putative selection with genes that may be related to adaptations to harsh environments (e.g., *AQP5*, *RAD50*, and *RETREG1*). We found genomic signatures of selective sweeps near key genes related to economically important traits, such as the milk production (e.g., *DGAT1*, *ABCG2*), growth (e.g., *XKR4*), and reproduction (e.g., *CSF2*). Our data point to candidate genes which should be included in future studies attempting to identify genes to improve the extant breeds and facilitate generation of commercial breeds that fit better into the environments of Russia and other countries with similar climates.

## Introduction

Recent advances in sequencing and genotyping technologies allow the identification of changes in genomes (‘selection signatures’) guided by positive selection in wild and domestic populations with a resolution and accuracy not previously achievable and at reasonable expense^1^. This has resulted in the construction of detailed selection signature maps for human populations^2–4^ and other species^5,6^. Domestic animals are of particular interest for this research because numerous traits have been affected by strong artificial selection for many generations, first during domestication and later during breed formation^7^. In addition, domesticated species (e.g., livestock) have been exposed to natural selection to adapt to the diverse environments into which they have been moved together with migrating human populations^8^ and during natural migrations prior to domestication^9^. Domestic cattle provide a good example of a species that has been domesticated at least twice in human history^10^, that has adapted to diverse environmental conditions ranging from Africa to Siberia^11^ and that has been under strong artificial selection to produce more than 1000 extant breeds^12^ exhibiting diverse levels of milk production, meat quality, feed efficiency and other economically important traits^13^. The main sources of modern cattle breed genetics are two *Bos* subspecies: *B*. *taurus taurus* and *B*. *taurus indicus* with some subsequent hybridisation events resulting in the descendant hybrid breeds being adapted to a large variety of environments^14,15^.

Genome studies of commercial cattle have allowed the identification of major genes and variants related to milk production (e.g., *DGAT1* and *ABCG2*), meat quality (e.g., *MSTN* and *TG*), feed efficiency (e.g., *ZIM2*) and coat colour (e.g., *KIT*, *KITLG*, and *MITF*). Similarly, a recent study of native African cattle pointed to candidate genes for hot climate adaptation, such as *HSPA4* and *SOD1*^16^ and a study of Chinese cattle has confirmed selection around well-established functional candidates (e.g., *MC1R*, *PAG1*, and *MYH* cluster) and has also revealed novel gene candidates^17^. These two examples demonstrate the power and the need for detailed studies of native/local breed genomes to reveal the genetic bases of economically important traits and adaptations to specific environments. Understanding the genetic backgrounds of selected traits within local environmental contexts may be useful in the design of new breeds that will combine the high productivity of developed commercial breeds with the adaptive alleles found in native breeds^18^.

As a step toward this goal for Eurasian cattle, we recently described the genetic history and population structure of the major Russian native cattle breeds relative to the commercial and native breeds previously collected from around the world^19^. Most of the Russian breeds have ancestry that is shared with European taurine cattle breeds, while a few breeds represented by a branch of the so-called Turano-Mongolian cattle (Kalmyk, Yakut, and Buryat) share ancestry with the Asian taurines including Japanese Black (Wagyu) and Korean Hanwoo. This lineage is diverged from the European taurine cattle and shares few common haplotypes with them excluding those that have been transferred by recent admixture events in some populations^19^. The history of Turano-Mongolian cattle breeds traces back to early postglacial times and some researchers have hypothesised the independent domestication of the Asian taurine cattle preliminarily supported by genetic^20^ and paleontological evidence^10^.

Herein, we present the results of scans for putative signatures of selection and adaptation in the most distinct populations of the Russian cattle breeds selected based on our previous results^19^. The local Russian breeds are presumably adapted to an environment that is characterized by a range of conditions: e.g., rich grazing pastures and high temperatures during the short summer period, but harsh weather conditions and short daytime during the long winter. Breeds such as the Yakut, which is native to the Eastern Siberia, can overwinter in the open with temperatures as low as −50 °C^21^. The unique adaptations found in the Russian breeds likely also include a biotic component characterized by the resistance to indigenous pathogen infections and parasites^22^. Despite this wide range of ecotype adaptation, reports on signatures of selection in Russian cattle breeds have been limited to sparse genotype data (54,000 Single Nucleotide Polymorphisms (SNPs)) and to only four breeds: Yakut, Yaroslavl, Kalmyk, and Ukrainian Grey analyzed together with six breeds from Northern Europe^23^ and breeds from China^24^. The authors reported selection in the region of the *ABCG2* gene related to milk production^25^ in the Yaroslavl breed and near virus resistance genes in the Yakut cattle which is supported by the known resistance of the breed to some viral infections^22^. Therefore, in this work we present analysis of signatures of selection for nine Russian native cattle breeds based on genotype data for ~139,000 SNPs combined with four additional breeds of European origin and two Asian taurine cattle breeds. We used hapFLK multipoint statistics considering the structure of haplotypes segregating within populations to reveal likely signatures of selection within three groups of related breeds and for individual breeds, de-correlated composite of multiple signals (DCMS) of selection combining H1 and H12 statistics, Tajima’s D, nucleotide diversity (Pi), and the fixation index (*F*_*ST*_) which have previously been shown to be more efficient than each of these statistics individually for the detection of candidate selection signatures^26–28^. Our results demonstrate that while the Russian cattle breeds share major common signatures of selection/adaptation with world breeds, they also exhibit unique putative signatures of selection that can be related to adaptation to local environment, e.g. the cold climates.

## Methods

### Genotype data for nine Russian cattle breeds

GeneSeek Genomic Profiler High-Density (GGP HD150K) genotype results for nine Russian cattle breeds (Table 1) were obtained from Yurchenko *et al*.^19^. For the Russian breeds of European origin, we used 139,378 genotypes which were combined with the corresponding whole-genome sequencing (WGS)-based data originating from additional breeds of European origin (Table 1; described below). For the Russian breeds of Asian origin, we combined the GGP HD150K genotypes with 770 K Illumina BovineHD array data for the Japanese Black and with the WGS-based genotype data for the same SNPs for the Hanwoo breed from Korea resulting in 105,099 autosomal SNPs.

**Table 1:** Breeds and breed groups.

Breed	No. samples	Type	Reference	Breed group
Kalmyk	23	beef	^19^	ASIA
Buryat	24	dual purpose	^19^	ASIA
Yakut	26	dual purpose	^19^	ASIA
Hanwoo	21	beef	^30^	ASIA
Japanese Black	19	beef	^14^	ASIA
Kazakh Whiteheaded	20	beef	^19^	EUR1
Kostroma	18	dual purpose	^19^	EUR1
Jersey	15	dairy	^29^	EUR1
Fleckvieh	25	dual purpose	^29^	EUR1
Kholmogory	30	dairy	^19^	EUR2
Bestuzhev	19	dual purpose	^19^	EUR2
Yaroslavl	18	dairy	^19^	EUR2
Black Pied	24	dairy	^19^	EUR2
Holstein	25	dairy	^29^	EUR2
Angus	25	beef	^29^	—
Average/Total	22/332	—	—	—

### SNP identification and filtering from WGS data

Sequence data for individuals from four European breeds (Holstein, Angus, Jersey, Fleckvieh) were obtained from the 1000 Bull Genomes project Run2^29^. Sequences for 23 Hanwoo samples were downloaded from the NCBI GenBank in fastq format^30^. The full sequence processing protocols are described in^31^. Briefly, fastq reads were trimmed for adaptors and quality (>20 phred), and filtered to have <3 Ns and to pass chastity. Reads were then aligned to the UMD3.1 bovine genome assembly using bwa mem^32^ and PCR duplicates were removed. Variants were called using SAMtools v. 1.3^33^ and filtered. High-quality SNPs were queried for their presence on the GGP HD150K array and genotypes from matched SNPs were extracted and merged with the GGP HD150K genotype data using PLINK^34 ^*--merge* command.

### Selecting animals and populations

**Figure 1:**
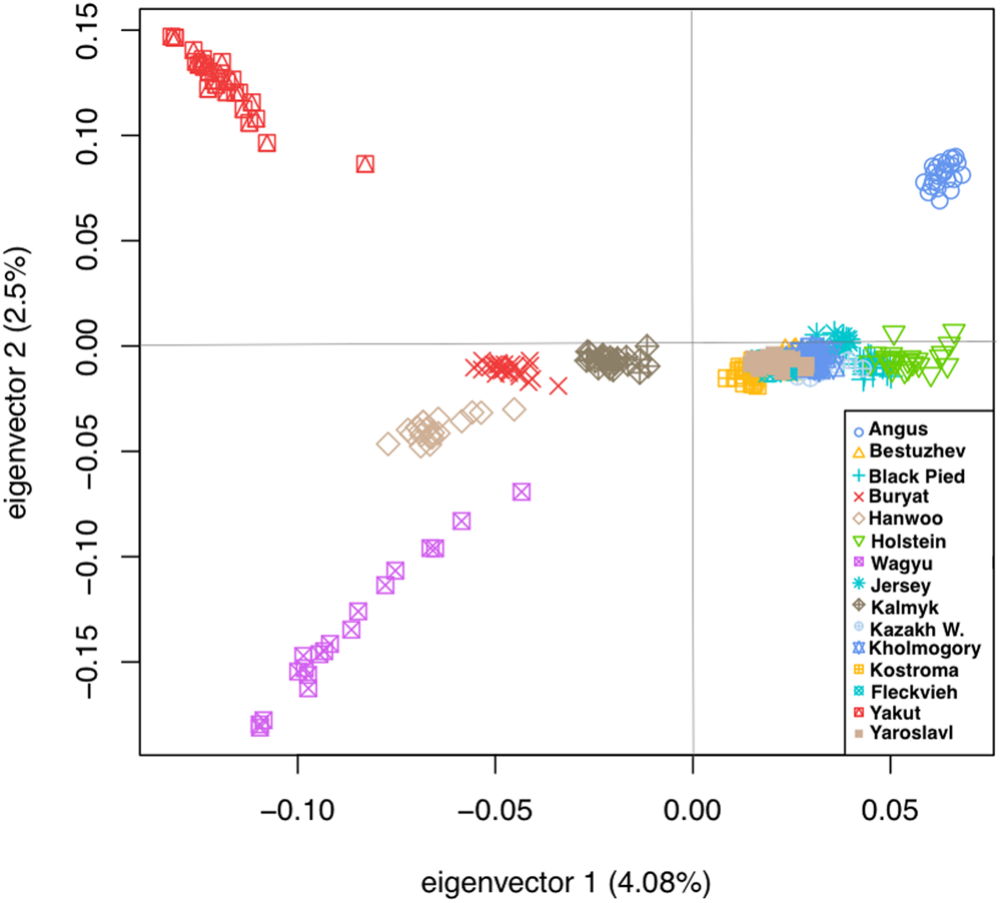
Principal component analysis of genotypes for nine native Russian breeds and additional cattle breeds of European and Asian origins used in the current study.

Russian breeds with fewer than 18 samples and those breeds exhibiting recent admixture with other breeds^19^ were removed from the analysis. Breed groups for the hapFLK analyses were selected based on the results of principal component analysis (PCA^35^) of the joint dataset of 15 cattle breeds (Fig. 1). The breeds formed three clusters that demonstrated no major evidence of recent admixture between the groups. Two of the clusters (EUR1 and EUR2) included Russian and foreign breeds of European ancestry while the last cluster (ASIA) included Russian and foreign Turano-Mongolian breeds (Fig. 1; Supplementary Fig. S1; Table 1). Outlier samples from each breed were identified from the PCA plots and were removed from the analyses.

To eliminate sample size bias for breeds with large numbers of genotyped individuals (e.g., >100; Holstein) we removed animals with a high relatedness to other members of the same breed. We calculated indexes of relatedness within Holstein using the PLINK --*genome* function. Pairs of animals with unusually high values for pairwise PI_HAT statistic sum had one member removed from the analysis.

### Identification of signatures of selection with hapFLK statistics

We performed a genome scan for signatures of selection within each group of breeds listed in Table 1 using a haplotype-based statistic (hapFLK)^36^. Because the hapFLK model assumes selection to be acting on ancestral SNP alleles we excluded rare SNPs with low minor allele frequencies (MAFs) from each of the three breed groups (MAF < 0.05). We also excluded poorly genotyped individuals (<95% of SNPs with genotypes), loci genotyped in <99% of samples, and SNPs on the sex chromosomes in PLINK using the commands: --*maf 0.05*, --*geno 0.01*, --*mind 0.05*, and --*chr 1–29* prior to performing the genome selection scans.

The hapFLK method takes the haplotype structure of the population into account. What was important for our dataset is that this method can account for population bottlenecks and migration. Reynold’s distances and a kinship matrix were calculated by the hapFLK program v.1.4^36^. For the hapFLK analysis, the number of haplotype clusters for each breed group was estimated with fastPhase^37^ and were set as –K 25, 35, 20 for the EUR1, EUR2, and ASIA breed groups, respectively. The expected maximum number of iterations was set to 30 for three groups. We used Yakut samples as outgroups to root the EUR1 and EUR2 population trees calculated by hapFLK and performed midpoint rooting for the ASIA set of breeds. Local Reynolds distances were calculated for selected regions using *local_reynolds*.*py* script and local population trees were then built with *local_trees* R script obtained from https://forge-dga.jouy.inra.fr/projects/hapflk/wiki/LocalTrees.

#### P-value calculation

For hapFLK the calculation of raw p-values was performed assuming that the selected regions represent only a small fraction of the genome^38^. The genome-wide distribution of hapFLK statistics could be modelled relatively well with a normal distribution except for a small fraction of outliers from potentially selected regions. Robust estimates of the mean and variance of the hapFLK statistic were obtained using the R MASS package, *rlm* function to eliminate influence of outlying regions following Boitard *et al.*^39^. This was done for each group (EUR1, EUR2, and ASIA). The hapFLK values were Z-transformed using these parameter estimates and p-values were calculated from the normal distribution in R. The R *qvalue* package was used to correct p-values for multiple testing^40^.

### Composite measure of selection

It has recently been demonstrated, that the application of the composite measures of selection significantly improves signal to noise ratio and increases the power for the location of signals of possible selection^27,28^, specifically in comparison to single statistics or their simple overlaps. We combined five genome-wide statistics including the fixation index (*F*_*ST*_^41^, haplotype homozygosity (H1^42^), modified haplotype homozygosity statistics (H12^42^), Tajima’s D index^43^ and nucleotide diversity (Pi^44^) in the de-correlated composite of multiple signals (DCMS) framework^27^ which was shown to be efficient for combining p-values from the individual test statistics. DCMS combines p-values produced by several statistics for each locus into a single measure considering the correlation between the statistics. The correlation matrix was calculated genome-wide and allowed the assignment of different weights to each statistic’s p-value depending on their genome-wide correlation.

#### Haplotype-based statistics

Genotypes were phased separately in the two breed groups from Asia (ASIA) and Europe (EUR1 + EUR2) using SHAPEIT2 software^45^ with 400 conditioning states (–*states 400*) and effective population size parameter equal to 5000 (–*effective-size 5000*) as a safe provisional estimate for our diverse dataset. We used recombination rate estimates from Ma *et al*.^46^ to correct for the variability in recombination rates along chromosomes.

Both the H1 and H12 statistics^42^ estimate levels of haplotype-based homozygosity in windows throughout the genome, but H12 calculates homozygosity using the frequencies of the first and the second most common haplotypes which allows more efficient detection of hard and soft selective sweeps which are common in populations of wild and domestic species^42,47,48^. To calculate the H1 and H12 statistics we used VCF files with phased genotypes that were converted to the specific format required by the *H12_H2H1.py* script (https://github.com/ngarud/SelectionHapStats) from Garud *et al*.^42^. We calculated the statistics individually for each breed using overlapping windows of 14 SNPs with a step size of one SNP.

#### Tajima’s D statistics

Tajima’s D statistics were calculated for the same overlapping 14 SNP windows using vcftools v. 0.1.15^49^ and the *–TajimaD* function. The intervals were formed based on the output of the H1 statistic analysis for the Asian and European breeds separately and then passed to the *bcftools* (*view*) software^33^ along with the breed-specific gzipped VCF file before being passed to the vcftools –TajimaD function. To reduce the time of calculation all the work was carried out in parallel mode with assistance of GNU PARALLEL^50^.

#### Nucleotide diversity (Pi)

Nucleotide diversity was calculated for each breed and chromosome separately using the *vcftools –site-pi* option. The produced values were then smoothed using the R *runmed* function with the window size of 31 SNPs (k = 31, endrule = “constant”).

#### Fixation index (*F*_*ST*_)

We calculated the *F*_*ST*_ index as a measure of population differentiation separately for the European (EUR1 + EUR2) and Asian breeds (ASIA). Within each of the groups, *F*_*ST*_ was calculated for each variant for each breed against the rest of the samples from the other breeds within the group using the PLINK --fst function. Negative *F*_*ST*_ values were converted in zeros and the statistics was smoothed for each chromosome using R *runmed* function in windows of 31 SNPs (k = 31, endrule = “constant”) to reduce noise.

#### De-correlated composite of multiple signals (DCMS)

To calculate the DCMS statistics for each of the breeds we combined the aforementioned statistics (H1, H12, Tajima’s D, Pi, and *F*_*ST*_) in a single spreadsheet by merging data on the basis of the SNP name. We next used the R MINOTAUR package^51^ to calculate genome-wide *P-*values based on fractional ranks for each statistic (*stat_to_pvalue* MINOTAUR function) with appropriate one-tailed tests (Pi and Tajima D – left-tailed; H1, H12, and *F*_*ST*_ – right-tailed). The covariance matrix between the different statistics was estimated based on 50,000 randomly sampled SNPs using the *CovNAMcd* function with alpha = 0.75. The DCMS statistic was calculated using the *DCMS* function and the precalculated covariance matrix among the statistics. The resulting DCMS statistics for each breed were examined for normality of their distributions and then fitted to the normal distribution using the robust fitting of linear model method implemented in the *rlm* R function of the MASS package^39^. The fitted DCMS statistics were then converted in p-values using the *pnorm* function (lower.tail = FALSE, log.p = FALSE) and the *P*-values were finally converted to the corresponding q-values using the *qvalue* R function^40^.

### Identification of chromosome intervals under selection and candidate genes

Bovine gene annotations from the bovine genome assembly build UMD 3.1 were downloaded from Biomart^52^. We then identified chromosome intervals and candidate genes predicted to have been subjected to selection. To locate the putative regions under selection we considered chromosome intervals with SNPs with adjusted p-values < 0.05 and boundaries of each interval were defined by the locations of the first flanking SNPs exhibiting adjusted p-values > 0.1. Within the selected intervals, genes were identified within 1σ value from the most significant SNP based on statistical values (DCMS or hapFLK) distribution similar to Fariello *et al*.^38^. This approach results in fewer candidate genes being reported for the “sharp” selection peaks while for the intervals with many SNPs exhibiting similar statistics values, larger numbers of genes were reported. Genes were also ranked based on their distance from the SNP with the highest statistics value in each region with larger ranks assigned to more distant genes. We visualized regions under putative selection using *circlize* R package^53^.

## Results

### Breed groups

The PCA analysis suggested the presence of two well-differentiated clusters of breeds in our dataset (Fig. 1) represented by the Asian Turano-Mongolian (ASIA: Yakut, Buryat, Kalmyk, Japanese Black, and Hanwoo) and European breeds matching our previous results^19^. In addition, in the hapFLK analysis, the European breed population was further subdivided into two sets of breeds (EUR1: Kazakh Whiteheaded, Kostroma, Jersey, Fleckvieh; and EUR2: Bestuzhev, Black Pied, Holstein, Kholmogory, Yaroslavl) matching the two major clusters of European breeds from Yurchenko *et al*.^19^ and supported by the PCA analysis (Supplementary Fig. S1). In total, 332 animals from 15 breeds (including nine Russian breeds) with a mean number of 22 individuals per breed were used in these analyses (Table 1).

The DCMS and hapFLK statistics for the Russian and foreign cattle breeds overlapped to some extent providing independent support for selected regions. However, because the hapFLK statistic detects probable signatures of selection within groups of breeds and the DCMS in our study was used to combine statistics within a breed, the hapFLK results could not be added to the DCMS framework. The hapFLK revealed additional regions under putative selection within groups of breeds missed by the DCMS while the DCMS was efficient in detecting shorter candidate regions.

### Composite measure of selection

The DCMS statistic was calculated for each SNP for each breed. After fitting of the normal distribution, calculation of p-values and correction for multiple testing we obtained 29 to 90 genomic intervals under putative selection per breed (q-value < 0.05) with a total of 953 regions detected across all breeds (with some overlaps between breeds; Supplementary Table S1). The size of the genomic regions putatively under selection varied from 1 bp to 4.80 Mbp with the average size of 242.46 Kbp. The number of genes within detected regions per breed ranged from 42 to 209.

### HapFLK

The total number of regions identified by the hapFLK analyses (46; Supplementary Table S1) was lower than found by the DCMS method. The largest number of hapFLK detected regions was observed in the EUR1 set (25), followed by the ASIA (12) and EUR2 (9) sets. The EUR1, EUR2, and ASIA sets all shared a common hapFLK interval (BTA5:13.2–27.00 Mbp, containing the *KITLG* gene), however, coordinates for sub-regions detected in the EUR1 and ASIA sets did not overlap. No other shared hapFLK regions were detected for any combination of the breed sets. Sizes of the putatively selected regions ranged from 484 Kbp to 15 Mbp with an average size of 2.8 Mbp.

### Candidate genes for adaptation of the Russian cattle breeds to environmental and climate challenges

**Figure 2:**
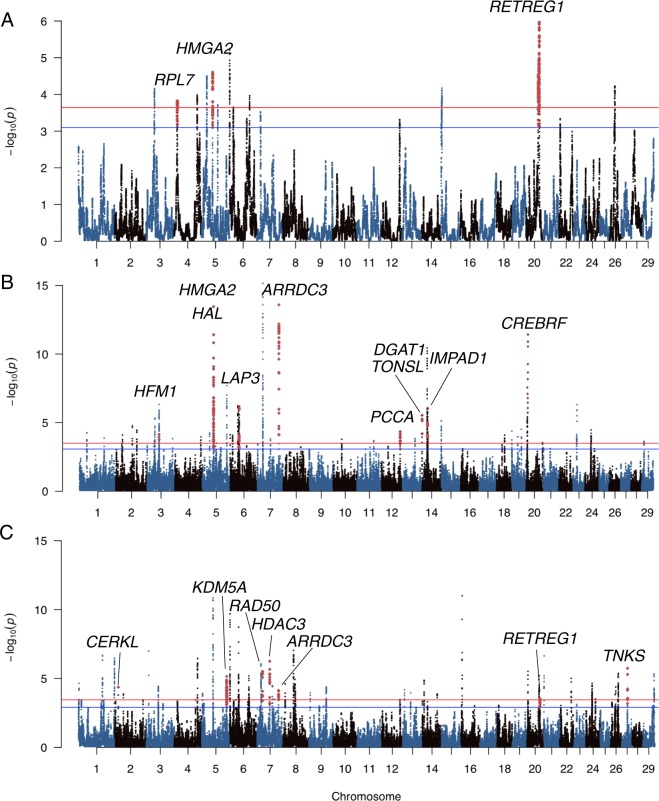
Manhattan plots for putative signatures of selection analysis in cattle breeds of Asian origin with names of candidate genes discussed in the text. **(A)** hapFLK results for the combined set of five breeds (Yakut, Buryat, Kalmyk, Hanwoo, and Japanese Black), **(B)** DCMS results for the Buryat cattle, **(C)** DCMS results for Yakut cattle. Blue line indicates q-value = 0.1 and the red line q-value = 0.05. Colored peak regions highlight positions of the genes indicated on the plots.

We investigated how adaptation to local environmental challenges including viral and parasite challenges and the cold climate could have shaped the genomes of the Russian native cattle breeds (Table 2). We found a region on BTA7:23.04–23.14 Mbp containing *RAD50* and reported by DCMS that appeared to have been likely selected in the Russian Kholmogory, Bestuzhev, Kalmyk, Yakut, and Yaroslavl breeds as well as in the Korean Hanwoo. RAD50 is a DNA repair protein, the component of MRN complex, that plays a central role in double-strand break repair and is a key gene in antiviral protection^54^ suggesting its potential role in the response of the local breeds to viral challenges. The cold-resistant Kholmogory cattle had a probable signature of selection in the region on BTA5:29.68–30.17 Mbp containing the aquaporin cluster (AQP@), including the top ranked in DCMS results *AQP5* gene previously reported as a cold/heat acclimation candidate due to its role in the formation of water channels^55^. *AQP5* has been under positive selection in yaks in response to adaptation to high altitudes^56^ characterised by low temperatures. Seven regions under putative selection were reported by hapFLK for the cold-adapted Yakut cattle, of which four were shared with the Japanese Black and two with Hanwoo cattle (Fig. 2; Supplementary Table S2). Top-ranked genes within the two intervals that were unique to the Yakut cattle according to the hapFLK haplotype local tree analysis (p-value < 0.05) were candidates for the reaction of organisms to cold exposure. The *RETREG1* is responsible for human pain insensitivity disorder caused by hereditary sensory and autonomic neuropathy (HSAN). Mutations in human *RETREG1* are responsible for HSAN Type 2^57^. HSAN Type 2 leads to inability to feel pain and temperature^58^. The Yakut cattle have a signature of selection in this region (p-value = 0.001) while Hanwoo possesses a suggestive signature of selection (p-value = 0.09) according to hapFLK and significant signature according to the DCMS analysis (Fig. 2; Table 2; Supplementary Fig. S2). The ribosomal large subunit protein 7 (*RPL7*) gene was shown to be cold-responsive in freeze tolerant frogs with higher protein levels found in the skin of cold-tolerant species compared to non-tolerant species and increased expression levels in muscles and brain under freezing conditions^59^. According to the hapFLK haplotype local tree analysis, this region could be under strong selection in the Yakut cattle (p-value = 0.00005) with a weak signal also observed in Hanwoo and Japanese Black (p-value = 0.05; Fig. 2; Supplementary Fig. S3). In addition, the Yakut cattle demonstrated a unique signature of putative selection in the genomic region containing the tankyrase (*TNKS*) gene (top ranked by the DCMS analysis in the region) that has been shown to be related to energy expenditure, feed intake and adiposity in mice^60^, suggesting its possible role in adaptation to variation in local environment feed availability and quality. The Yakut cattle also had a signature of selection in the region of the keramide kinase like gene (*CERKL)* expressed in the retina, which possesses variants responsible for retinitis pigmentosa in humans, associated with light stress response and protection of photoreceptor cells^61^. This putative signature of selection could be related to the adaptation of the Yakut cattle to the light regime above the Polar circle.

**Table 2:** Genes in regions predicted to be under putative selection in Russian cattle breeds.

Chr*	Region start (Mbp)	Region end (Mbp)	Most significant SNP q-value	Breed/Group	No. genes in the region	Method	Candidate gene (rank)	Function
1	28.402	28.899	0.002273827	Kazakh Whiteheaded	2	DCMS	*FKBP2* (2)	Milk
2	15.069	15.083	0.012500925	Yakut	1	DCMS	*CERKL* (1)	Light stress
2	73.106	73.393	0.017181621	Yaroslavl	1	DCMS	*GLI2* (1)	Weight, growth
3	9.348	9.751	0.004315819	Bestuzhev	13	DCMS	*COPA* (3)	Coat colour
3	9.348	9.722	0.001173551	Black Pied	10	DCMS	*COPA* (1)	Coat colour
3	9.386	9.529	0.046081992	Holstein	2	DCMS	*COPA* (1)	Coat colour
3	52.040	52.213	0.001189865	Hanwoo	1	DCMS	*HFM1* (1)	Reproduction
3	52.075	52.231	0.010464401	Holstein	1	DCMS	*HFM1* (1)	Reproduction
3	52.148	52.225	0.009368275	Black Pied	1	DCMS	*HFM1* (1)	Reproduction
3	52.153	52.187	0.018393593	Buryat	1	DCMS	*HFM1* (1)	Reproduction
3	52.174	52.267	0.000324907	Kalmyk	1	DCMS	*HFM1* (1)	Reproduction
4	14.188	15.448	0.040960871	ASIA	6	hapFLK	*RPL7* (1)	Acclimation
4	107.007	107.085	0.043707068	Kalmyk	3	DCMS	*TRPV5* (1)	Milk fever
5	13.271	27.026	5.53E-08	EUR2	71	hapFLK	*KITLG* (4)	Coat colour
5	16.357	21.155	0.001789011	Yaroslavl	13	DCMS	*KITLG* (9)	Coat colour
5	17.154	20.486	0.000398061	EUR1	8	hapFLK	*KITLG* (1)	Coat colour
5	18.335	19.335	4.47E-05	Bestuzhev	1	DCMS	*KITLG* (1)	Coat colour
5	18.335	18.966	0.001068985	Kazakh Whiteheaded	1	DCMS	*KITLG* (1)	Coat colour
5	29.684	30.174	0.011869128	Kholmogory	10	DCMS	*AQP5* (3)	Acclimation
5	47.752	48.287	0.033364267	ASIA	1	hapFLK	*HMGA2* (1)	Growth
5	48.017	48.308	4.53E-10	Buryat	1	DCMS	*HMGA2* (1)	Growth
5	48.017	48.506	7.46E-11	Kalmyk	1	DCMS	*HMGA2* (1)	Growth
5	48.045	48.263	0.001471807	Hanwoo	1	DCMS	*HMGA2* (1)	Growth
5	48.094	48.514	4.68E-06	Bestuzhev	1	DCMS	*HMGA2* (1)	Growth
5	48.155	48.280	0.018053667	Japanese Black	1	DCMS	*HMGA2* (1)	Growth
5	48.185	48.270	0.001526729	Kostroma	1	DCMS	*HMGA2* (1)	Growth
5	60.624	60.624	0.048486709	Buryat	1	DCMS	*HAL* (1)	Milk
5	75.685	75.731	0.037717028	Yaroslavl	1	DCMS	*CSF2RB* (1)	Milk
5	107.338	108.490	0.004370697	Yakut	18	DCMS	*KDM5A* (2)	Milk
6	37.076	39.850	0	EUR1	18	hapFLK	*NCAPG* (1), *LCORL* (2), *LAP3* (6), *ABCG2* (11)	Growth, Milk
6	37.855	38.000	1.04E-06	Kalmyk	2	DCMS	*ABCG2* (1)	Milk
6	37.902	37.948	0.027503049	Fleckvieh	1	DCMS	*ABCG2* (1)	Milk
6	37.983	38.188	0.006537637	Kostroma	3	DCMS	*ABCG2* (1)	Milk
6	38.579	38.656	0.001009406	Kholmogory	3	DCMS	*LAP3* (2)	Milk
6	38.580	38.616	0.000578482	Buryat	3	DCMS	*LAP3* (1)	Milk
6	38.731	38.924	7.26E-07	Jersey	3	DCMS	*NCAPG* (1), *LCORL* (3)	Growth
6	38.735	38.931	0.000382606	Kazakh Whiteheaded	3	DCMS	*NCAPG* (1), *LCORL* (2)	Growth
6	38.924	39.100	0.006598304	Fleckvieh	1	DCMS	*LCORL* (1)	Growth
6	38.924	39.023	0.03478244	Yaroslavl	1	DCMS	*LCORL* (1)	Growth
6	59.928	74.936	0	EUR1	63	hapFLK	*KIT* (4)	Coat colour
6	68.059	72.063	5.67E-07	Kazakh Whiteheaded	26	DCMS	*KIT* (4)	Coat colour
6	69.440	72.476	1.13E-06	Fleckvieh	10	DCMS	*KIT* (4)	Coat colour
6	71.240	71.959	0.000166418	Yaroslavl	3	DCMS	*KIT* (2)	Coat colour
7	23.035	23.183	0.001289242	Kholmogory	1	DCMS	*RAD50* (1)	Antimicrobial protection
7	23.078	23.107	0.046379711	Bestuzhev	1	DCMS	*RAD50* (1)	Antimicrobial protection
7	23.078	23.261	3.43E-08	Kalmyk	3	DCMS	*IL5 (1)*, *IRF1* (2), *RAD50* (3)	Antimicrobial protection
7	23.113	23.228	2.35E-05	Hanwoo	2	DCMS	*RAD50* (1), *IL5* (2)	Antimicrobial protection
7	23.136	23.498	3.66E-06	Bestuzhev	4	DCMS	*IRF1* (1), *IL5* (3)	Antimicrobial protection
7	23.136	23.216	0.00265416	Yakut	2	DCMS	*IL5* (1), *RAD50* (2)	Antimicrobial protection
7	23.144	23.144	0.037325408	Yaroslavl	1	DCMS	*RAD50* (1)	Antimicrobial protection
7	23.591	23.695	0.005771811	Holstein	1	DCMS	*CSF2* (1)	Reproduction
7	23.613	23.699	0.000131001	Black Pied	1	DCMS	*CSF2* (1)	Reproduction
7	23.683	23.698	0.002131539	Yaroslavl	1	DCMS	*CSF2* (1)	Reproduction
7	23.691	23.696	0.028601712	Kazakh Whiteheaded	1	DCMS	*CSF2* (1)	Reproduction
7	54.361	54.735	0.000687902	Yakut	6	DCMS	*HDAC3* (5)	Brown adipose tissue
7	93.194	93.360	2.19E-07	Kalmyk	1	DCMS	*ARRDC3* (1)	Adipose tissue
7	93.206	93.307	3.83E-10	Buryat	1	DCMS	*ARRDC3* (1)	Adipose tissue
7	93.208	93.276	0.01177964	Fleckvieh	1	DCMS	*ARRDC3* (1)	Adipose tissue
7	93.211	93.307	2.37E-07	Hanwoo	1	DCMS	*ARRDC3* (1)	Adipose tissue
7	93.214	93.276	0.010240366	Kostroma	1	DCMS	*ARRDC3* (1)	Adipose tissue
7	93.215	93.224	0.031503554	Jersey	1	DCMS	*ARRDC3* (1)	Adipose tissue
7	93.242	93.282	0.017682048	Yakut	1	DCMS	*ARRDC3* (1)	Adipose tissue
8	0.264	0.503	0.04889224	Kholmogory	1	DCMS	*ANXA10* (1)	Reproduction
8	79.681	79.759	0.027035565	Yaroslavl	1	DCMS	*NTRK2* (1)	Antimicrobial protection
8	88.219	88.589	0.002222339	Black Pied	1	DCMS	*SYK* (1)	Brown adipose tissue
8	88.420	88.465	0.027629081	Kholmogory	1	DCMS	*SYK* (1)	Brown adipose tissue
12	44.257	44.839	0.001059257	Bestuzhev	1	DCMS	*KLHL1* (1)	Milk
12	80.782	81.010	0.012757213	Buryat	1	DCMS	*PCCA* (1)	Milk
12	80.803	81.092	0.010062102	Yaroslavl	1	DCMS	*PCCA* (1)	Milk
12	80.962	81.085	0.037912516	Japanese Black	1	DCMS	*PCCA* (1)	Milk
13	57.572	57.636	0.021930321	Kazakh Whiteheaded	1	DCMS	*EDN3* (1)	Coat colour
14	1.489	1.893	0.032866336	Kazakh Whiteheaded	28	DCMS	*TONSL* (2), *DGAT1* (20)	Milk
14	1.651	1.869	0.001653056	Buryat	11	DCMS	*TONSL* (1), *DGAT1* (11)	Milk
14	1.696	1.893	0.038247912	Kholmogory	13	DCMS	*DGAT1* (1)	Milk
14	23.258	23.269	0.02863779	Yaroslavl	1	DCMS	*NPBWR1* (1)	Reproduction
14	24.419	25.135	1.02E-08	Black Pied	8	DCMS	*TMEM68* (2), *XKR4* (8), *PLAG1* (5)	Feed intake, Growth, Weight
14	24.421	24.501	0.00525187	Kazakh Whiteheaded	1	DCMS	*XKR4* (1)	Weight
14	24.426	25.135	1.50E-06	Kholmogory	8	DCMS	*TMEM68* (2), *PLAG1* (5), *XKR4* (7)	Feed intake, Growth, Weight
14	24.431	24.434	0.047221525	Kalmyk	1	DCMS	*XKR4* (1)	Weight
14	24.512	24.556	0.032774966	Hanwoo	1	DCMS	*XKR4* (1)	Weight
14	24.699	25.010	0.000703648	Bestuzhev	3	DCMS	*TMEM68* (2)	Feed intake
14	24.714	24.909	1.24E-06	Kalmyk	3	DCMS	*TMEM68* (3)	Feed intake
14	25.447	25.849	5.88E-06	Kazakh Whiteheaded	1	DCMS	*IMPAD1* (1)	Growth
14	25.460	25.617	4.79E-10	Holstein	1	DCMS	*IMPAD1* (1)	Growth
14	25.497	25.667	0.002016879	Angus	1	DCMS	*IMPAD1* (1)	Growth
14	25.497	25.546	0.014138416	Kostroma	1	DCMS	*IMPAD1* (1)	Growth
14	25.499	25.541	0.015989917	Jersey	1	DCMS	*IMPAD1* (1)	Growth
14	25.507	25.530	0.000738867	Buryat	1	DCMS	*IMPAD1* (1)	Growth
15	35.637	36.167	0.013241977	Black Pied	5	DCMS	*NUCB2* (1)	Feed intake, Body temperature, Growth
16	36.496	36.581	0.045474804	Bestuzhev	1	DCMS	*RGS7* (1)	Domestication, Acclimation
20	4.046	4.102	0.00256072	Jersey	1	DCMS	*SH3PXD2B* (1)	Growth, meat
20	4.050	4.084	0.000859753	Bestuzhev	1	DCMS	*SH3PXD2B* (1)	Growth, meat
20	4.067	4.144	0.003706131	Black Pied	1	DCMS	*SH3PXD2B* (1)	Growth, meat
20	4.067	4.084	0.019181385	Holstein	1	DCMS	*SH3PXD2B* (1)	Growth, meat
20	4.593	4.784	0.0029386	Kholmogory	4	DCMS	*CREBRF* (1)	Growth
20	4.597	4.730	0.0004249	Bestuzhev	3	DCMS	*CREBRF* (1)	Growth
20	4.597	4.791	0.0145958	Jersey	4	DCMS	*CREBRF* (3)	Growth
20	4.628	4.782	0.0002944	Holstein	3	DCMS	*CREBRF* (1)	Growth
20	4.660	4.783	2.94E-05	Hanwoo	3	DCMS	*CREBRF* (1)	Growth
20	4.673	4.759	0.0010948	Buryat	2	DCMS	*CREBRF* (1)	Growth
20	4.692	4.782	0.0125848	Black Pied	2	DCMS	*CREBRF* (1)	Growth
20	4.692	4.761	6.08E-05	Kalmyk	2	DCMS	*CREBRF* (1)	Growth
20	31.912	32.005	0.019009149	Black Pied	1	DCMS	*GHR* (1)	Milk, Growth
20	32.098	32.460	0.032750776	Bestuzhev	1	DCMS	*GHR* (1)	Milk, Growth
20	52.303	57.558	0.009549042	ASIA	8	hapFLK	*RETREG1* (1)	Acclimation
20	56.431	56.876	0.043882656	Yakut	3	DCMS	*RETREG1* (2)	Acclimation
20	56.683	56.890	0.000763043	Hanwoo	2	DCMS	*RETREG1* (2)	Acclimation
24	60.758	62.644	0.000623541	EUR2	15	hapFLK	*BCL2* (1)	Reproduction
27	24.577	24.803	0.001749199	Yakut	1	DCMS	*TNKS* (1)	Feed intake, Adipose tissue
28	21.176	26.909	0.00551179	EUR2	30	hapFLK	*SIRT1* (2)	Antimicrobial protection
28	24.039	24.885	0.01399301	Yaroslavl	6	DCMS	*SIRT1* (2)	Antimicrobial protection
28	35.332	35.883	0.008902995	Yaroslavl	2	DCMS	*SFTPD* (2)	Antimicrobial protection

^*^For a full list of selected regions and genes see Supplementary Table S1.

Brown adipose tissue is an organ that provides energy to protect animals from hypothermia^62^. The key gene in this process is a mitochondrial uncoupling protein 1 (*UCP1*)^62^. We found several genes known to influence expression of *UCP1* and that are directly involved in the regulation of adiposity in regions under putative selection in the Russian cattle breeds. The histone deacetylase 3 (*HDAC3*) gene, that is required to activate brown adipose tissue enhancers to ensure thermogenic aptitude^63^ was found in a reported region in the Yakut cattle, while the adipocyte arrestin domain-containing 3 protein (*ARRDC3*), that regulates the expression of *UCP1* in white adipose tissue^64^, was found in the region to be under probable selection in four Russian breeds, Hanwoo, Jersey, and Fleckvieh. This is suggestive of its potential role in variation in economically important traits. On the other hand, only the Kholmogory and Black-Pied breeds possessed selection signals in a region that included spleen tyrosine kinase (*SYK*) which is involved in brown adipocyte differentiation and is known to affect the expression of *UCP1*^65^. The Yaroslavl cattle expressed a strong signal of putative selection (q-value < 0.01) in the genomic region that includes *SFTPD* that encodes lung surfactant protein D (SP-D) which contributes to lung defense from inhaled microorganisms and was previously found to be under positive selection in human populations adapted to high altitudes^66^. The Black Pied cattle had been exposed to putative selection in a region including the top-ranked *NUCB2* gene that encodes Nesfatin-1 which influences the regulation of body temperature and food intake^67^. In cattle, genetic variants in *NUCB2* have been shown to be associated with growth traits in three native Chinese breeds^68^. A signature of likely selection in the Bestuzhev breed was detected near *RGS7* that has been shown to be related to the differentiation of neurological function between dogs and wolves and, in humans, its expression in central noradrenergic neurons increases in response to chronic cold exposure^69^.

### Morphological traits and adaptations

**Figure 3:**
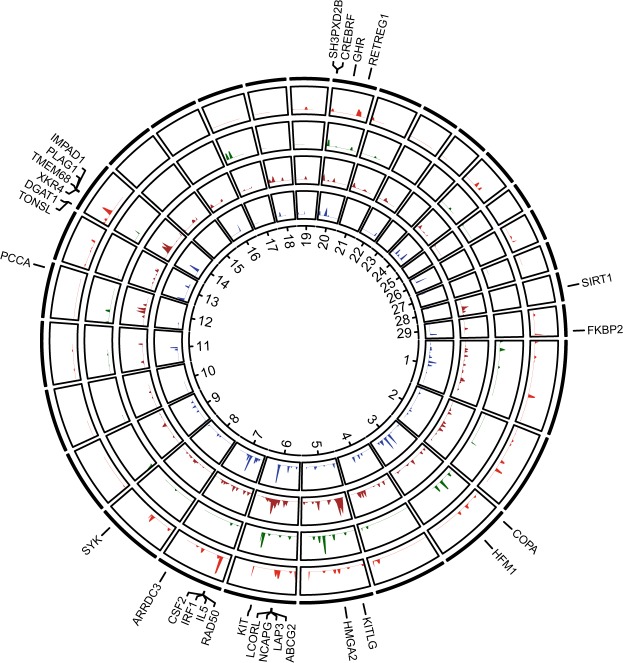
Circos plot showing signatures of putative selection in cattle genomes. In blue are signatures of selection detected in the European breeds of non-Russian origin, in brown are signatures of selection in the Russian cattle breeds of European origin, in green are Asian breeds of non-Russian origin while in red are Russian breeds of Asian origin. Numbers correspond to cattle autosomes. Candidate genes found in putatively selected regions in at least two breeds or in both the DCMS and hapFLK analyses reported in Table 2 are placed on the outer circle. For a full list of all regions and genes see Supplementary Table S1.

Of the 999 genomic intervals (953 from DCMS and 46 from hapFLK) under putative selection, 66.7% overlapped with regions previously predicted to have been under selection in cattle^70^ (Supplementary Table S1). Among these previously detected regions, strong signals of differentiation were obtained in the regions containing well known candidate genes related to morphology, adaptation, and domestication (e.g., *KITLG*, *KIT*, *EDN3*, and *COPA*), growth and feed intake (*XKR4*, *TMEM68*, *LCORL*, *NCAPG*, *HMGA2*, *IMPAD1*, and *GLI2*), reproduction (*CSF2*, *BCL2*, *ANXA10*, and *NPBWR1*), and milk traits (*DGAT1*, *GHR*, *ABCG2*, *GLI2*, *LAP3*, *TRPV5*, *FKBP2*, and *PCCA*; Fig. 3).

#### Morphology and domestication

Domestication is often associated with changes in the coat colour of domesticated populations^71^. Consistent with previous findings in livestock and other domestic species, strong signals of putative selection in Russian breeds of European and Asian origin were detected by the hapFLK analyses in the genomic regions that include the genes *KIT* and *KITLG*, both of which contribute to coat colour in a variety of species^72,73^. Interestingly, in the region including *KITLG* a long interval under putative selection (BTA5: 13.27–27.03 Mbp (13.76 Mbp)) was reported by hapFLK in the EUR2 breed set, while in the EUR1 set the overlapping region was shorter (BTA5:17.15–20.49 (3.34 Mbp)). On the other hand, the DCMS analysis detected multiple putative signatures of selection within the 13.76 Mbp interval on BTA5 in different breeds with Bestuzhev and Kazakh Whiteheaded being the only Russian breeds that had a narrow signal near the *KITLG* gene, while the Black Pied and Yaroslavl had several signals of putative selection near other genes suggesting that this locus could contain multiple sequences subjected to selection (Supplementary Table S1). Consistent with this finding, in the Asian breeds a 1.60 Mbp overlapping region was revealed (BTA5: 21.97–23.57 Mbp) which did not, however, include the *KITLG* (Supplementary Table S1). A similar pattern was observed near the *KIT* gene. The hapFLK analyses identified a region exposed to putative selection in the EUR1 breed set on BTA6:59.93–74.94 Mbp (15.01 Mbp). However, the DCMS analysis detected multiple shorter intervals within this region with *KIT* being found in putatively selected regions in the Yaroslavl, Kazakh Whiteheaded, and Fleckvieh breeds. Two more coat colour-related genes (*EDN3*, *COPA*) were top-ranked by DCMS among the signatures of selection detected in the Kazakh Whiteheaded (*EDN3*), Black Pied (*COPA*), and Holstein (*COPA*) breeds. *EDN3* promotes the differentiation and proliferation of melanocytes^74^ and has previously been found in one of 12 genomic regions associated with the UV-protective eye area pigmentation phenotype in the Fleckvieh whiteface breed^75^. Since our Kazakh Whiteheaded individuals did not have well-developed eye area pigmentation but all had whiteface phenotypes, we can speculate about the *EDN3* be directly involved with formation of the whiteface phenotype itself. Additionally, the Black Pied, Bestuzhev, and Holstein breeds had putative signatures of selection near the coatomer protein complex, subunit alpha (*COPA*) gene, which is known to be related to pigment synthesis. A missense mutation within this gene is completely associated with dominant red coat phenotype in Holstein cattle^76^.

#### Growth and feed intake

A putative signature of selection has previously been detected in a region on BTA14 containing the *XKR4* gene that is associated with birth weight in Nelore cattle^77^ as well as with feed intake and average daily gain in cattle^78,79^. In the Black Pied and Kholmogory the selected interval was relatively wide containing also the *TMEM68* gene previously associated with feed intake^79^ and *PLAG1* that is associated with body size, weight and reproduction in cattle^80^, while in the Kazakh Whiteheaded the region was smaller containing only *XKR4*. Kalmyk cattle may possess two separate putatively selected regions in this interval, one near *XKR4* and the other near *TMEM68*. The *LCORL-NCAPG* interval on BTA6 has been demonstrated to be associated with growth traits in cattle (average daily gain, muscle development^81^ and carcass composition^82^). In our analysis, the Kazakh Whiteheaded and Jersey demonstrate relatively wide signals in this region (~190 Kb) containing both genes with *NCAPG* being the top-ranked by DCMS. The Yaroslavl and Fleckvieh had shorter intervals with the *LCORL* gene being the top-ranked. A putative signature of selection was reported by both the hapFLK in the ASIA set and DCMS in the Buryat, Kalmyk, Bestuzhev, Kostroma, Hanwoo, and Japanese Black breeds near *HMGA2* (high mobility group protein A2), a transcription factor that regulates genes involved in cell differentiation and growth. This is a key gene associated with growth in cattle^83^, human height^84^, body size in dogs^85^, and horses^86^, weight in mice and carcass traits in pigs^87^. We identified a likely signature of selection in the region of inositol monophosphatase domain containing 1 (*IMPAD1*) on BTA14 reported by DCMS in the Kazakh Whiteheaded, Kostroma, and Buryat breeds. *IMPAD1* plays a role in the bone-cartilage system with mutations leading to severe growth retardation in humans^88^. This region has also previously been found to be under selection in Canchim^89^ and Brahman cattle^90^. DCMS also detected a signature of putative selection in the region containing *GLI2* (a member of the Gli gene family, which encode transcription factors) in the Yaroslavl cattle. This gene has previously been associated with bovine weight^91^ and growth traits in pigs^92^.

#### Reproduction

A strong signal of putative selection was detected near the colony stimulating factor 2 (*CSF2*) in Black Pied, Yaroslavl, and Kazakh Whiteheaded breeds with the narrowest signal (5 Kbp) observed in the Kazakh Whiteheaded. *CSF2* is important for embryonic development in cattle^93,94^ due to its role in inhibiting apoptosis^95^. Another gene involved in cattle oocyte/blastocyst apoptosis (*BCL2*^96^), was identified near the most significant SNP in the BTA24: 60.76–62.64 Mbp interval found for the EUR2 breed set. *ANXA10* on BTA8 has previously been associated with embryonic mortality in Japanese Black through a large chromosomal deletion^97^ and was found in a putatively selected region in Kholmogory cattle. *NPBWR1* within a likely selected region detected in the Yaroslavl breed was previously associated with fertility traits in Nelore cattle^98^.

#### Milk production traits

A putative signature of selection detected in the Kazakh Whiteheaded, Buryat, and Kholmogory breeds at BTA14:1.49–1.89 Mbp contains a major gene controlling milk fat content (*DGAT1*^99^). The dairy Kholmogory cattle had the narrowest selected region (196 Kbp) with *DGAT1* being the top-ranked gene by DCMS. Another major gene affecting milk yield and content, *ABCG2*^25^, was found in a 2.77 Mbp region detected by hapFLK on BTA6:37.08–39.85 Mbp in the EUR1 set of breeds. However, this interval also includes other candidate genes such as *LCORL* and *NCAPG*. The DCMS approach further divided this region into smaller intervals with *ABCG2* being the top-ranked gene in regions detected for the Kalmyk and Kostroma breeds. On the other hand, in the Buryat cattle, the most significant SNP in the same region was found near the *LAP3* gene, also associated with milk production traits^100^ and identified as the most likely candidate affecting direct calving ease in the Piedmontese breed^101^. A gene with a pleotropic effect, *GHR*, affecting protein and milk yield in dairy cattle^102^ and growth^103^ was found in an interval detected by the DSMC analysis in the Black Pied and Bestuzhev breeds. The transient receptor potential cation channel subfamily V member 5 gene (*TRPV5*) associated with hypocalcemia in cattle and with milk fever^104^ was found in a 77-Kbp selected interval of BTA4 reported for the Kalmyk cattle. The Kazakh Whiteheaded breed had a reported region on BTA1 which contained *FKBP2*. The *FKBP2* gene was previously associated with milk protein yields and percentage in Holstein GWAS studies^105^. In the Kazakh Whiteheaded and Buryat we identified large regions (~400 Kbp and ~200 Kbp respectively) on BTA14 which contained the gene *TONSL* previously identified as a putative candidate for cattle milk traits in a genotype-by-sequencing association study^106^. Among other genes related to milk production traits are the *CSF2RB* gene located in a putatively selected region in the Yaroslavl breed, associated with milk production in a large Jersey and Holstein cohort and differentially expressed in mammary gland^107^; *KLHL1* previously shown to be associated with milk yield and lactation persistence in the Chinese Holstein breed^108^ and in a region predicted to be under probable selection in the Bestuzhev breed; *HAL* in a region predicted to be under putative selection in Buryat cattle and previously associated with the milk traits in Chinese Holstein^109^; *KDM5A* located in a region reported for the Yakut breed and identified as a key regulator of the fatty acid levels in the milk of Brown Swiss cattle^110^; *PCCA* located in a region reported for Buryat and Yaroslavl breeds and previously associated with metabolic adaptation to divergent milk production performance^111^.

#### Other candidate genes

Among the other genes in narrow putatively selected intervals found in multiple Russian breeds, we identified several that could be functional candidates for important cattle traits. Among these are the ATP-dependent DNA helicase homolog (*HFM1*), associated with ovarian insufficiency in humans^112^ and found in regions reported for the Buryat, Kalmyk, and Black Pied Russian breeds, as well as in Hanwoo and Holstein. Mutations within *SH3PXD2B*, located in regions predicted to be under putative selection in the Bestuzhev and Black Pied Russian breeds, are associated with skeletal abnormalities in humans^113^, whereas in pigs this gene has previously been associated with intramuscular fat content^114^ suggesting its role in growth and meat related traits. Another candidate gene found in regions predicted to be likely selected in the Buryat, Kalmyk, Black Pied, Holstein, and Hanwoo breeds is *CREBRF* that has been associated with obesity, weight and height in humans^115–117^ and identified as a key regulator of endometrial function in goats^118^.

We finally searched the list of putatively selected regions for genes related to disease resistance. The strongest candidates included the interferon regulatory factor 1 (*IRF1*) which induces inflammatory responses in macrophages^119^ and is known to be associated with mycobacterium susceptibility in humans^119^ and mice^120^. *IRF1* was the top-ranked gene in the DCMS results in a 362 Kbp putatively selected region reported for the Bestuzhev breed and second-ranked in Kalmyk cattle. The same region also contains interleukin 5 (*IL5*), top-ranked for Kalmyk and Yakut cattle and known to be involved in the immune response to mycobacterium infection in humans^121^. Another gene previously found to be associated with tuberculosis susceptibility in wild boars^122^, neurotrophic tyrosine kinase receptor, type 2 (*NTRK2*) was top-ranked in a 77.7 Kbp region of BTA8 reported for the Yaroslavl breed. The Yaroslavl breed also had a probable signature of selection near the sirtuin 1 (*SIRT1*) gene, a nicotinamide adenine dinucleotide (NAD^+^)-dependent deacetylase expressed in monocytes/macrophages. *SIRT1* is involved in the modulation of lung myeloid cells in mycobacterium-infected mice. Moreover, myeloid cell-specific Sirt1 knockout mice showed increased susceptibility to mycobacterium infection^123^.

## Discussion

We report the first comprehensive genome-wide autosomal analysis of putative signatures of genomic selection in the genomes of nine Russian native cattle breeds utilizing comparative data originating from six additional breeds of European and Asian origins. Integration of our genotype data^19^ with genotype- and sequence-generated SNPs from additional breeds allowed us to differentiate between shared signatures of selection and those that might be unique to breed(s) of Russian origin. Our data suggest that while the Russian cattle breeds share a significant fraction of probably selected regions with other breeds, they also possess some unique signatures of adaptation/selection that might be related to adaptive responses to local environments. Therefore, on the one hand, our results prove the power of using native breed genomes to reveal novel candidate signatures of selection formed as adaptive responses to specific environments, while on the other hand point to selective sweeps near key genes controlling important traits suggesting that selective breeding utilizing established genetic markers could be applied to further improve some of the local Russian breeds.

As expected, the composite measurement of statistics (combining SNP and haplotype-based individual breed statistics) resulted in a larger number of intervals being reported as putatively selected compared to the haplotype-based hapFLK approach based on breed group data. However, 30% of hapFLK regions were not detected the DCMS analysis and 83% of the DCMS regions were not found in by the hapFLK analyses, suggesting that the hapFLK approach was conservative in identifying selected regions involving long haplotypes while the DCMS analysis was more sensitive in detecting shorter selected regions.

Our results demonstrate that the Yakut cattle which are adapted to survive above the Polar circle^23^ possess signatures of putative selection that contain the genes *RETREG1* and *RPL7* which may contribute to the adaptation of this breed to its harsh environment. However, suggestive signatures of selection near these genes were also found in other Turano-Mongolian breeds (Japanese Black and Hanwoo), indicating that some differentiation in these intervals could be present in the ancestral Turano-Mongolian pool of animals making these animals more suitable for future adaptation to extreme cold conditions of Northern Russia than other taurine cattle. Life above the Polar Circle also requires adaptation to specific light regime and the ability to defend against novel parasites, viral and bacterial infections. Consistent with such requirements, multiple genes related to these processes were located in genomic regions predicted to be under putative selection in the Yakut cattle. Other Russian cattle breeds, which live in less extreme environments but that are exposed to cold temperatures especially during the winter also had reported regions near genes that are related to protection against viral infections, known candidate genes for cold acclimation (e.g., the Kholmogory) and other genes that are known to change their expression levels in response to exposure to cold in other species. We observed multiple genes involved in brown adipose tissue development as being in genomic regions that are under putative selection in Russian cattle breeds, including *HDAC3* and *SYK*. Brown adipose tissue is an organ involved in non-shivering thermogenesis, suggesting that these genes may be involved in adaptation to cold climates. However, caution is required in interpreting these results as we observed that some of these genes were also found in regions predicted to have been selected in other cattle breeds, including the European taurines. Adipose tissue is also an important component of meat, suggesting that these genes are related to meat production traits which have been artificially selected.

In addition to genes that might be related to adaptation to local environment, the genomes of the Russian cattle breeds possess signatures of putative selection in regions containing genes that are related to domestication and morphology. One region contains the *KITLG* gene that is known to be responsible for the roan coat colour phenotype in cattle. In our study it was predicted to be under selection in the European cattle breeds but the overlapping selected region in the Asian cattle did not include *KITLG*, suggesting that other genes in this region could be under selection in the Asian taurine cattle. We also failed to identify strong signal in the region containing *KIT* (several coat colour phenotypes) in the Asian cattle breeds, but it expressed strong signal in the European cattle. This might suggest that different mechanisms of coat colour determination exist in the two groups of breeds or that the statistical power of analysis in Asian taurines was not high enough to reveal signatures of selection in this region.

We observed a strong relationship between the beef type of the breed and known genes related to meat quality and growth located in the detected putative signatures of selection; such as *XKR4* in Kazakh Whiteheaded and Kalmyk beef breeds, *NCAPG*, *LCORL* in Kazakh Whiteheaded and *IMPAD1* in Kazakh Whiteheaded, Buryat, and Kostroma dual purpose breeds. Another growth-related gene, *HMGA2* was located in a region predicted to be under selection in the Kalmyk beef breed and the Bestuzhev and Kostroma dual purpose breeds. On the other hand, genes related to milk production were located in regions reported for all types of breeds. For instance, the *DGAT1* was reported not only for the dairy Kholmogory breed and dual purpose Buryat cattle but also for the beef Kazakh Whiteheaded. However, only the Kholmogory breed had *DGAT1* as a top-ranked gene in a narrow interval, while the other two breeds had much wider regions under putative selection and *DGAT1* was low in rank (11 and 20). Another major gene related to milk production, *ABCG2* was located in a region predicted to be under selection in the dual-purpose Kostroma and beef Kalmyk cattle. There is some evidence, however, that Kalmyk cattle were selected for their unique high dry-matter content of the milk^124^.

In conclusion, results of the first scan for signatures of putative selection/adaptation in genomes of nine Russian native cattle breeds demonstrate that their genomes contain multiple intervals likely subjected to selection, some of which appear to be related to adaptation to harsh (cold) environments. This analysis demonstrates the importance and power of studying local breeds that may not be as productive as the commonly used commercial breeds world-wide, but hold a real promise for decoding mechanisms of environmental adaptation to be utilized in genetics-guided improvement of productive multinational breeds.

## Electronic supplementary material

Supplementrary Information

Supplementary Table 1

## Data Availability

Data available from the Dryad Digital Repository: 10.5061/dryad.fr88s7f.
