# Supplementary material for: Scans for signatures of selection in Russian cattle breed genomes reveal new candidate genes for environmental adaptation and acclimation
Source: Sci Rep. 2018 Aug 28;8:12984. doi: 10.1038/s41598-018-31304-w (PMC6113280; doi:10.1038/s41598-018-31304-w)
Supplement: Supplementary file 1 — Supplementrary Information [file 41598_2018_31304_MOESM1_ESM.pdf]

Scans for signatures of selection in Russian cattle breed genomes reveal new candidate genes for environmental adaptation and acclimation

Andrey A. Yurchenko, Hans D. Daetwyler, Nikolay Yudin, Robert D. Schnabel, Christy J. Vander Jagt, Vladimir Soloshenko, Bulat Lhasaranov, Ruslan Popov, Jeremey F. Taylor, Denis M. Larkin

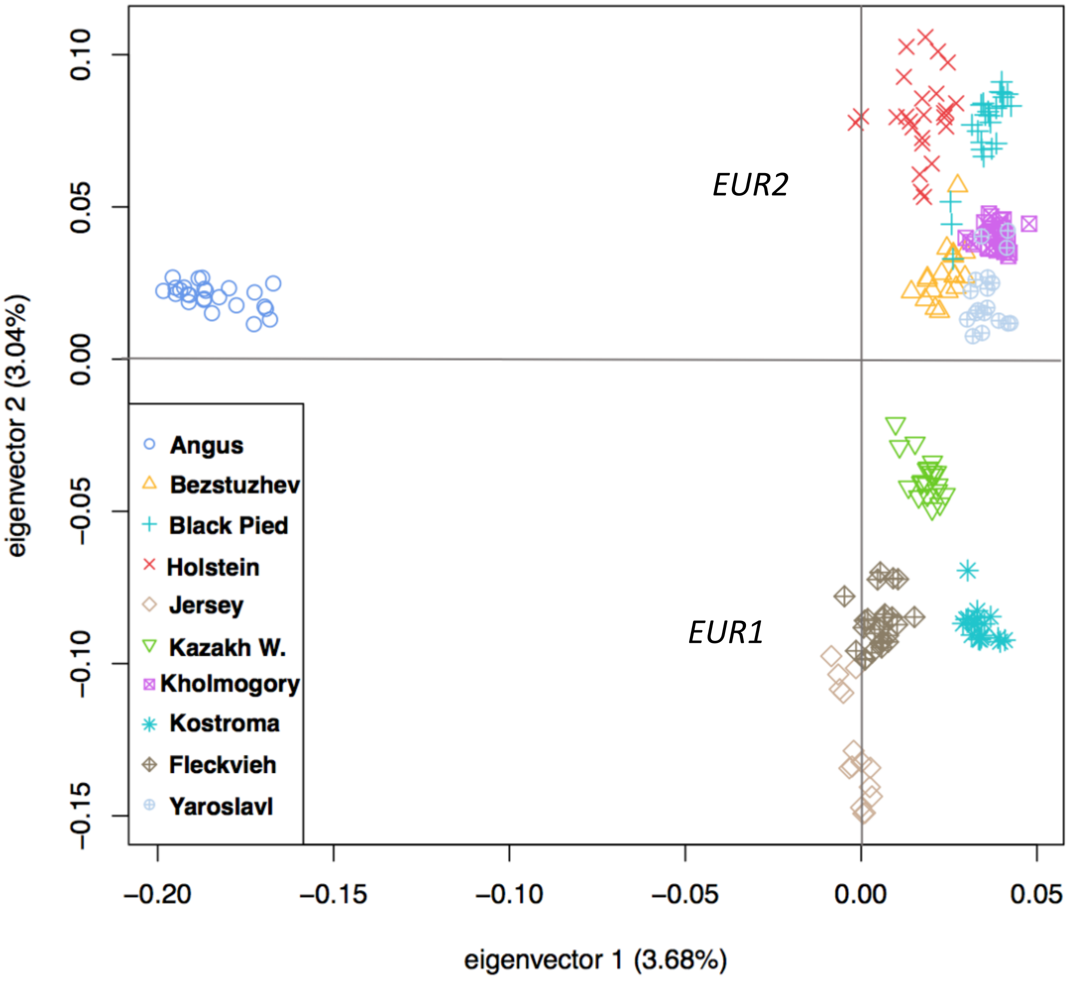

Supplementary Figure S1. Principal component analysis of genotypes from native Russian breeds and additional cattle breeds of European origins. Two clusters of European breeds used in hapFLK analyses are indicated by ovals and named EUR1 and EUR2.

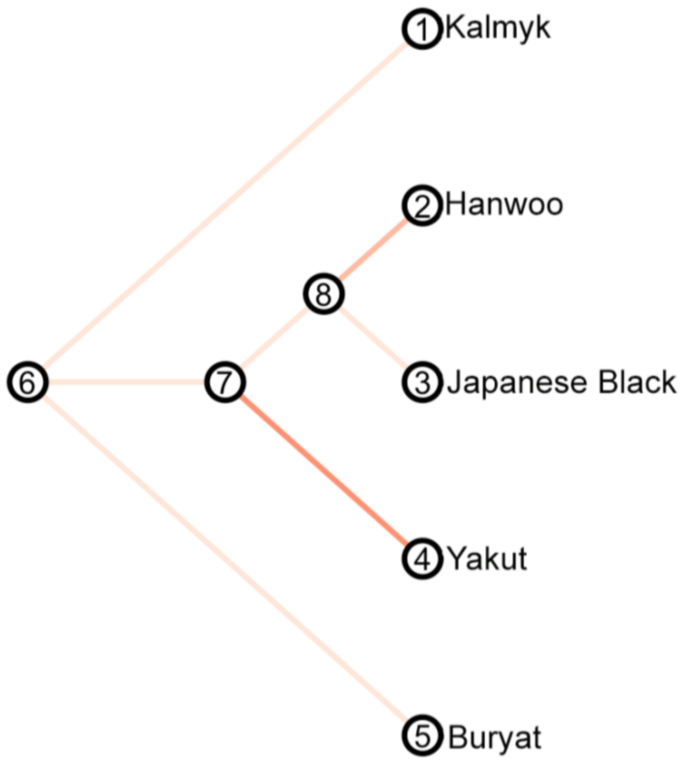

Supplementary Figure S2. Haplotypes local tree for the interval on chr20:52.30-57.56 Mbp showing a significant signature of selection on the Yakut cattle branch (p-value = 0.001) and a suggestive signature on the Hanwoo branch (p-value = 0.09).

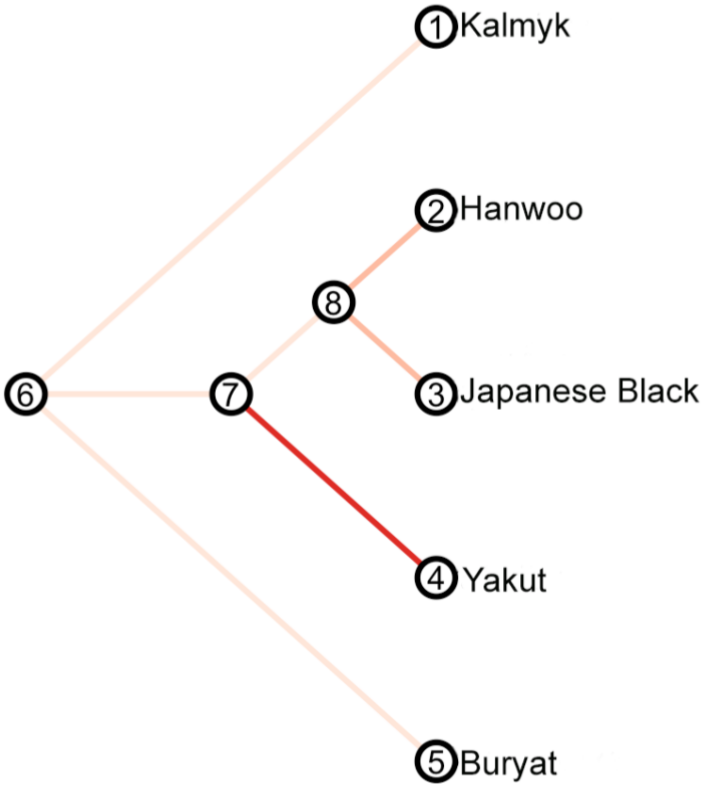

Supplementary Figure S3. Haplotypes local tree for the interval on chr4:14.19-15.45 Mbp showing a significant signature of selection on the Yakut cattle branch (p-value = 0.00005) and suggestive signatures on the Hanwoo and Japanese Black branches (p-values = 0.05).

Supplementary Table S1. Regions predicted to be under putative selection and genes in these regions in Russian and additional cattle breeds of European and Asian origins (in a separate Excel file).

Supplementary Table S2. Regions under putative selection detected by hapFLK analysis in Asian cattle breeds and breeds possessing selective signatures according to haplotype local tree analysis (p-value <0.05).

Chr	Start	End	Size (bp)	Method	Selected in breeds
3	36,134,005	37,327,971	1,193,967	hapFLK	Japanese Black
4	14,187,799	15,447,776	1,259,978	hapFLK	Yakut
4	101,346,553	102,230,528	883,976	hapFLK	Yakut, Japanese Black
5	21,965,045	23,568,941	1,603,897	hapFLK	Hanwoo, Japanese Black
5	47,752,157	48,286,719	534,563	hapFLK	Hanwoo, Japanese Black
5	70,124,916	70,767,676	642,761	hapFLK	Hanwoo
6	1,054,786	1,950,128	895,343	hapFLK	Yakut, Japanese Black
6	16,844,998	17,328,796	483,799	hapFLK	Yakut, Hanwoo
6	87,916,172	88,421,804	505,633	hapFLK	Yakut, Hanwoo, Japanese Black, Buryat
15	2,556,267	4,679,251	2,122,985	hapFLK	Japanese Black
20	52,303,230	57,558,340	5,255,111	hapFLK	Yakut
26	23,339,772	24,918,578	1,578,807	hapFLK	Yakut, Japanese Black
